# Pregnancy Outcomes in Liver and Cardiothoracic Transplant Recipients: A UK National Cohort Study

**DOI:** 10.1371/journal.pone.0089151

**Published:** 2014-02-19

**Authors:** Olaa Mohamed-Ahmed, Cathy Nelson-Piercy, Kate Bramham, Haiyan Gao, Jennifer J. Kurinczuk, Peter Brocklehurst, Marian Knight

**Affiliations:** 1 National Perinatal Epidemiology Unit, Nuffield Department of Population Health, University of Oxford, United Kingdom; 2 Division of Women’s Health, King’s College London, Women’s Health Academic Centre, King’s Health Partners, United Kingdom; 3 Obstetric Medicine, Guy’s and St Thomas’ NHS Foundation Trust, London, United Kingdom; 4 Institute for Women’s Health, University College London, United Kingdom; UCL Institute of Child Health, University College London, United Kingdom

## Abstract

**Introduction:**

There are an increasing number of reports of pregnancy in transplant recipients but many questions remain regarding the effect of the transplant on pregnancy outcome, the pregnancy on the graft and the medication on the fetus. The majority of studies reporting outcomes in transplant recipients have focused on women with kidney transplants, and have included retrospective, voluntary registries or single centre studies.

**Methods:**

The UK Obstetric Surveillance System (UKOSS) was used to prospectively identify all pregnant women with a liver or cardiothoracic transplant in the United Kingdom, between January 2007 and January 2012. Data were collected on demographics, transplant characteristics, immunosuppression regimens, antenatal care, maternal, graft and neonatal outcomes. In an exploratory analysis, we tested for associations between “poor fetal outcome” and medications used before or during pregnancy.

**Results and conclusions:**

We report 62 pregnancies in 56 liver transplant recipients and 14 pregnancies in 14 cardiothoracic transplant recipients (including 10 heart, three lung and one heart-lung recipient). Liver transplant recipients, in comparison to cardiothoracic, had similar livebirth rates (92% vs. 87%) but better fetal outcomes (median gestational age 38 weeks vs. 35 weeks; median birthweight 2698 g vs. 2365 g), fewer caesarean deliveries (47% vs. 62%), fewer maternal intensive care (ICU) admissions (19% vs. 29%) and fewer neonatal ICU admissions (25% vs. 54%). Nine women (12%) were taking mycophenolate mofetil at conception, which was associated with adverse fetal outcomes. Pregnancy in transplant recipients may have successful outcomes, but complication rates are high, emphasising the role of pre-conception counselling and further research into the long-term effect on maternal and graft survival rates.

## Introduction

Over the past 50 years, more than 14,000 women with solid organ transplants have had pregnancies, worldwide [1]. The majority of studies on pregnancy outcomes in women with transplants have included only women with renal transplants, with information obtained from national, retrospective, voluntary registries, with the only currently active registry being the National Transplantation Pregnancy Registry (NTPR), in the United States of America (USA) [2]–[4].

An international conference on reproduction and transplantation highlighted the need for prospective observational studies [5], and recognised that many unanswered questions remain. For the practicing clinician, further information is required regarding the effect of the transplant on pregnancy, the effect of pregnancy on the graft and the impact of medications on the fetus, particularly in non-renal transplant recipients [6].

The aim of this study was to use the United Kingdom Obstetric Surveillance System (UKOSS), which collects data on rare disorders in pregnancy [7], to conduct a national, prospective cohort study of pregnancy outcomes in liver and cardiothoracic transplant recipients.

## Methods

We aimed to identify all pregnant women in the United Kingdom (UK), between January 2007 and January 2012, who had previously undergone liver or cardiothoracic transplantation.

**Figure 1:**
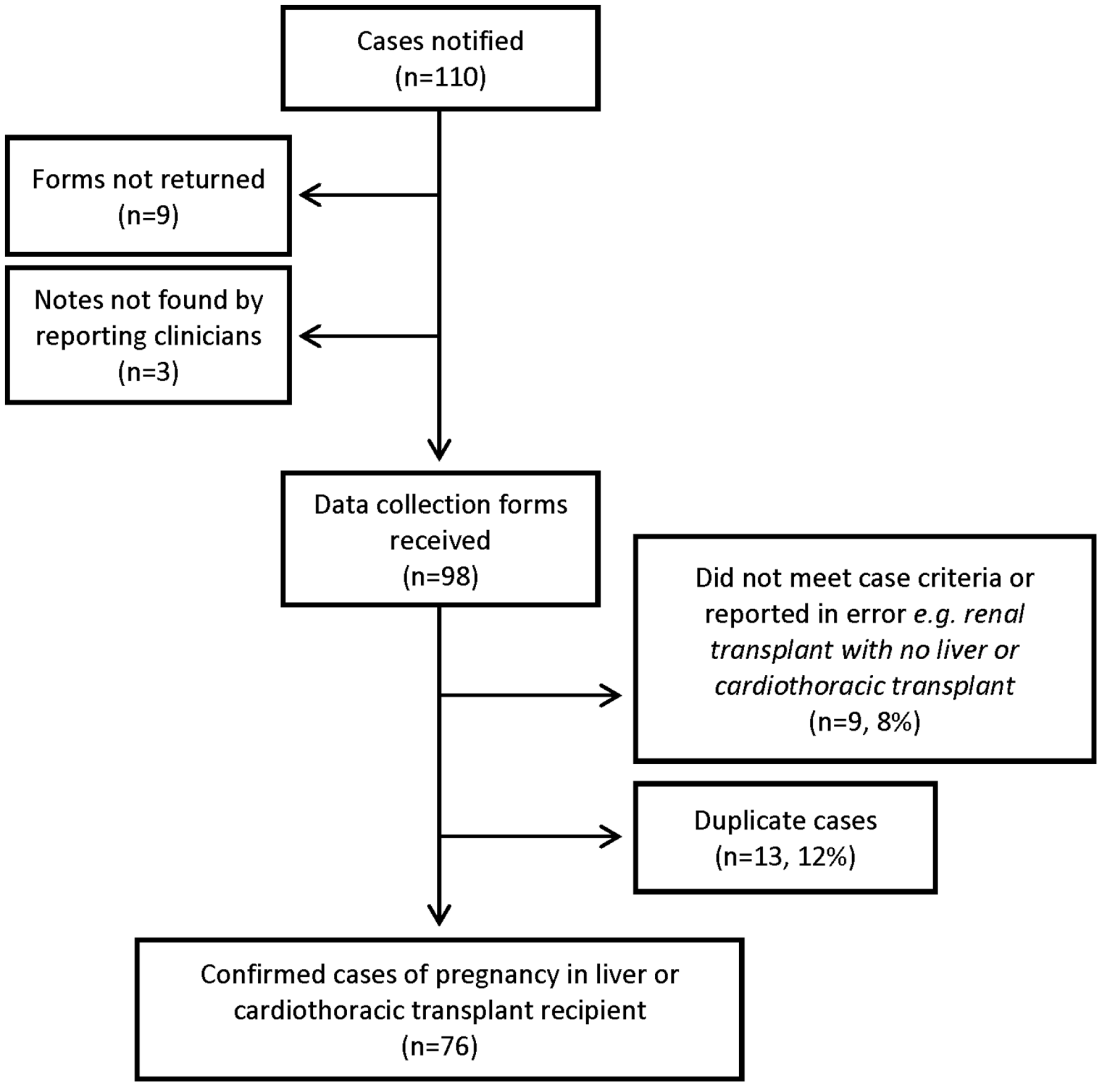
Case reporting and completeness of data collection.

The UKOSS methodology has been described in detail elsewhere [7]. In brief, nominated clinicians in each consultant-led maternity unit in the UK were sent a case notification card each month and asked to report all cases. They were also asked to return cards indicating a “nil report” in order to distinguish no cases of liver and cardiothoracic transplant recipients from a lack of response. Reporting clinicians were then asked to complete data collection forms to provide information about cases (Figure 1).

We collected data on demographics, transplant characteristics, immunosuppression regimens, antenatal care, and maternal, graft and neonatal outcomes. All data collected were anonymous and the entire cohort of women giving birth in the UK was included. As the study spanned five years, year of birth, height and the organ transplanted were used to identify successive pregnancies in the same recipient.

Continuous variables were summarized as means (standard deviations), or medians (inter-quartile or entire ranges) for skewed data. Categorical variables were summarized as frequencies (percentages).

Small-for-gestational age was calculated by comparing birthweight to revised British 1990 birth centiles [8], using LMSgrowth software [9]. As data were not collected on the sex of infants born to the transplant recipients, a best-case scenario was generated assuming all infants were female and worst-case scenario assuming all infants were male. Any infants below the 10^th^ centile were considered to be small-for-gestational age.

Poor fetal outcome was defined as any pregnancy resulting in a stillbirth, miscarriage, very low birthweight (<1500 g), small-for-gestational age (<10^th^ centile, best-case scenario), congenital anomaly, neonatal unit admission and very preterm birth (<32 weeks). This was used to generate a categorical variable which was tested for association with medications used before or during pregnancy. To allow for non-independence of multiple pregnancies from the same women, logistic regression with cluster analysis was used to generate odds ratios, p-values and 95% confidence intervals.

All statistical analyses were carried out using STATA 11 SE software (StataCorp LP, College Station, TX).

### Ethics Statement

The UK Obstetric Surveillance System general methodology (ref: 04/MRE02/45) and this study (ref: 06/MRE02/78) were approved by the London Multicentre Research Ethics Committee.

## Results

All 228 hospitals in the UK with obstetrician-led maternity units participated in the study (100% of eligible units), with data collection from January 2007 until February 2012. Nineteen hospitals ceased reporting cases during the study period, because the admitting units had closed. Case ascertainment is presented in Figure 1.

### Patient Characteristics

We identified 62 pregnancies in 56 liver transplant recipients and 14 pregnancies in 14 cardiothoracic transplant recipients, including 10 heart, three lung and one heart-lung recipient.

The demographic, maternal and transplant characteristics of the population are presented in Table 1.

**Table 1:** Demographic, maternal and transplant characteristics of liver and cardiothoracic transplant recipients.

Demographic Characteristics	Liver transplant cohort (n = 62)	Cardiothoracic transplant cohort (n = 14)
**Maternal age (years)**		
<20	3 (5)	0 (0)
20–34	45 (73)	11 (79)
≥35	14 (23)	3 (21)
**Ethnic group** 1		
White	44 (80)	12 (92)
Non-White	11 (20)	1 (8)
**Socio-economic status**		
Managerial/Professional	17 (33)	2 (15)
Non-managerial/Other	26 (51)	6 (46)
Unemployed	8 (16)	5 (38)
**Smoking status**		
Smoked during pregnancy	16 (27)	3 (21)
Did not smoke during pregnancy	43 (73)	11 (79)
**Body mass index**		
Normal (<25)	34 (57)	11 (79)
Overweight (25–29)	18 (30)	1 (7)
Obese (≥30)	8 (13)	2 (14)
**Multiple pregnancy**		
No	62 (100)	13 (93)
Yes	0 (0)	1 (7)
**Parity**		
0	34 (55)	10 (71)
1	18 (29)	4 (29)
2+	10 (16)	0 (0)
**Transplant to conception interval**		
Less than 1 year	2 (3)	0 (0)
1–2 years	5 (8)	0 (0)
2–5 years	16 (26)	3 (21)
More than 5 years	39 (63)	11 (79)

Data are shown as *n* (%), with percentages referring to complete data only.

1 Reported for 70 transplant women, rather than 76 pregnancies, as this characteristic will not have changed with repeated pregnancies.

Women with a liver transplant had a median age of 30 years at pregnancy (range 18–39 years), median age of 21 years at first transplantation (range 2–36 years, 34% below age 18) and a median transplant to conception interval of 6.5 years (range 4 months to 20 years). Seven women conceived within two years of liver transplantation, with two occurring within the first year. The most common indications for transplantation (Table 2) were acute liver failure (secondary to drug toxicity), biliary atresia, metabolic diseases, seronegative and autoimmune hepatitides.

**Table 2:** Indication for transplantation in liver transplant recipients (n = 56) and cardiothoracic transplant recipients (n = 14).

Category	Indication	Number (%)
**Liver transplant recipients**	**Acute liver failure**	**15 (27)**
	Paracetamol	7 (13)
	Other (ecstasy, sulfasalazine, viral)	8 (14)
	**Biliary atresia**	**8 (14)**
	**Cirrhosis**	**13 (23)**
	Seronegative/autoimmune hepatitis	11 (20)
	Other (alcohol, amyloid)	2 (3)
	**Metabolic disease**	**13 (23)**
	Wilson’s disease	8 (14)
	Other (tyrosinaemia, alpha-1 antitrypsin deficiency)	5 (9)
	**Other** (Budd-Chiari syndrome, primary sclerosing cholangitis, cystic fibrosis, malignancy)	**7 (13)**
**Cardiothoracic transplant recipients**	**Bronchiectatic disease**	**3 (21)**
	Cystic fibrosis	2 (14)
	Obliterative bronchiectasis	1 (7)
	**Cardiomyopathies**	**6 (43)**
	Viral	3 (21)
	Dilated	2 (14)
	Non-infective	1 (7)
	**Congenital heart disease and primary pulmonary hypertension**	**5 (36)**

Women with a cardiothoracic transplant had a median age of 26 at delivery (range 20–38 years), median age of 21 at first transplantation (range 4–33 years, 43% below age 18) and a median transplant to conception interval of 8 years (range 2–16 years), reflecting the burden of congenital disease, with almost half (n = 6) transplanted for congenital heart disease and cystic fibrosis (Table 2). No women conceived within two years of receiving a cardiothoracic transplant.

### Management

Of the 76 transplant recipients, 45 received antenatal care in the usual hospital for their area of residence (59%). Of those who received care at another hospital, 28 (37%) were referred because of their underlying medical condition.

### Immunosuppressants and Medication during Pregnancy

Tacrolimus was the most commonly used immunosuppressant in both groups of recipients (n = 58, 76%), followed by prednisolone (38%) and azathioprine (36%), as shown in Table 3. Nine women were taking mycophenolate mofetil (MMF) at conception, with three continuing MMF throughout the pregnancy (doses ranging from 500 to 2000 mg per day); one woman took sirolimus throughout her pregnancy.

**Table 3:** Medications taken before or during pregnancy.

Drugs	Liver transplant cohort (n = 62)	Cardiothoracic transplant cohort (n = 14)
**Immunosuppressants**		
Azathioprine	20 (32)	7 (50)
Cyclosporine	12 (19)	5 (36)
Prednisolone	24 (39)	5 (36)
Mycophenolate mofetil	7 (11)	2 (14)
Tacrolimus	49 (79)	9 (64)
Sirolimus	1 (2)	0 (0)
**Antihypertensives**		
ACE inhibitors and angiotensin receptor blockers	1 (2)	2 (14)
Calcium-channel blockers	4 (6)	1 (7)
Other antihypertensives	5 (8)	6 (43)
**Other**		
Aspirin	8 (13)	3 (21)
Dyspepsia drugs e.g. omeprazole, ranitidine	10 (16)	2 (14)
Anticoagulants	3 (5)	1 (7)

Data are shown as *n* (%), with percentages referring to complete data only.

Three women took ACE inhibitors or angiotensin receptor blockers at conception and 11 women took aspirin at conception.

### Fetal Outcomes

Fetal outcomes are reported in Table 4 and Table 5. There were 70 live births (91% of all pregnancies), and the live birth proportion was similar between the cardiothoracic and liver recipients. There were two stillbirths, five miscarriages/terminations and no neonatal deaths.

**Table 4:** Birth outcomes for 77 fetuses born to liver and cardiothoracic transplant recipients^1^.

Birth outcome	Entire cohort (n = 77)
Livebirth^2^	70 (91)
Termination of pregnancy for deteriorating maternal condition	1 (1)
First or second trimester miscarriage	4 (5)
Stillbirth	2 (3)

^1^Data have been grouped for confidentiality purposes, due to small numbers.

^2^Includes 57 livebirths to women with liver transplants and 13 livebirths to women with cardiothoracic transplants.

**Table 5:** Fetal outcomes^1^ in liver (n = 57) and cardiothoracic transplant recipients (n = 13)*.

	Liver transplant cohort n (%)	Cardiothoracic transplant cohort n (%)
**Apgar score at 5 minutes**		
More than 7	56 (98)	11 (85)
Less than 7	1 (2)	2 (15)
**Gestational age at delivery**		
Less than 32 weeks	0 (0)	2 (15)
32–37 weeks	24 (42)	5 (38)
More than 37 weeks	33 (58)	6 (46)
**Birthweight**		
1000–1499 g	1 (2)	1 (8)
1500–1999 g	6 (11)	3 (23)
2000–2499 g	14 (25)	3 (23)
More than 2500 g	36 (63)	6 (46)
**Small-for-gestational age**		
Best-case scenario	9 (16)	1 (8)
Worst case scenario	12 (21)	3 (23)
**Congenital anomaly**	0 (0)	0 (0)
**Neonatal unit admission**	14 (25)	7 (54)
**Infant breastfed**		
Yes	36 (63)	8 (62)
No	13 (23)	2 (15)
Not known	8 (14)	3 (23)

Data are shown as *n* (%), with percentages referring to complete data only.

1 Denominator includes all live births,

*including one multiple pregnancy in cardiothoracic cohort.

The median gestational age for live births to women with liver transplants was 38 weeks, compared to 35 in the cardiothoracic cohort (42% vs. 54% born before 37 weeks’ gestation). The median birthweight in the liver cohort was 2698 g (range 1115–3995 g), with 37% classified as low birthweight (<2500 g), compared to 2364 g (range 1480–3420 g) in the cardiothoracic cohort and 54% classified as low birthweight.

Thirty percent of neonates were admitted to a neonatal unit, with 54% (n = 7) of the cardiothoracic cohort compared with 25% of the liver cohort (n = 14). Our “best-case scenario” estimated only one small-for-gestational age infant in the cardiothoracic cohort (8%), compared to 9 (16%) in the liver cohort; “worst-case scenario” estimated three (23%) and 12 (21%), respectively.

In our exploratory analysis, MMF had a statistically significant association with poor fetal outcomes (p = 0.04, data not shown), with seven of nine women, who received it prior to or during pregnancy, experiencing adverse outcomes (odds ratio 5.31, 95% confidence interval 1.05–26.96, Table 6). No other immunosuppressant was associated with adverse fetal outcomes.

**Table 6:** Association of fetal outcomes with medications taken before or during pregnancy, in liver and cardiothoracic transplant recipients (n = 77)^1^.

	Good fetaloutcome (n = 43)	Poor fetaloutcome^2^ (n = 34)		Total(n = 77)		Odds ratio(95% confidence interval)
**Immunosuppressants^3^**						
Azathioprine	16 (37)	12 (35)		28 (36)		0.92 (0.36–2.23)
Cyclosporine	10 (23)	8 (24)		18 (23)		1.02 (0.37–2.78)
Prednisolone	18 (42)	12 (35)		30 (39)		0.76 (0.30–1.94)
Mycophenolate mofetil	2 (5)	7 (21)		9 (12)		5.31 (1.05–27.0)
Tacrolimus	33 (77)	25 (74)		58 (75)		0.84 (0.31–2.30)
Sirolimus	0 (0)	1 (3)		1 (1)		Insufficient data
**Anti-hypertensives** 4						
ACE inhibitors and ARBs	2 (5)	1 (3)		3 (4)		0.62 (0.05–7.27)
Calcium-channel blockers	4 (9)	1 (3)		5 (6)		0.30 (0.03–2.86)
Other	5 (12)	6 (18)		11 (14)		1.63 (0.45–5.91)
**Other** 4						
Aspirin	10 (23)	2 (6)		12 (16)		0.21 (0.05–0.78)
Dyspepsia drugs	7 (16)	5 (15)		12 (16)		0.89 (0.27–2.84)
Anticoagulants	3 (7)		2 (6)		5 (6)	0.83 (0.19–3.63)

Data are shown as *n* (%), with percentages referring to complete data only, except for the last column which gives odds ratios with 95% confidence intervals in parentheses.

^1^Denominator refers to all pregnancies, including one multiple pregnancy, but with cluster analysis for 70 women as six women had repeated pregnancies.

^2^Poor fetal outcome was defined as any pregnancy resulting in a stillbirth, miscarriage, very low birthweight (<1500 g), small-for-gestational age (<10^th^ centile, best-case scenario), congenital anomaly, neonatal unit admission and very preterm birth (<32 weeks).

3 Refers to medications taken before and/or during pregnancy.

4 Refers to medications taken before pregnancy.

ACE = Angiotensin-converting enzyme inhibitor; ARB = Angiotensin II receptor antagonist.

Women receiving aspirin appeared less likely to have a poor fetal outcome (p = 0.02, data not shown), with an odds ratio (OR) of 0.21 (95% confidence interval 0.05–0.78).

Sixty-three percent of women were breastfeeding their infants at discharge (n = 44).

### Maternal Outcomes and Complications

Maternal outcomes are presented in Table 7. One cardiac transplant recipient was delivered at 30 weeks’ gestation for deteriorating graft function, was admitted to intensive care and died 12 days later, with post-mortem biopsy confirming acute rejection. Two other women (one liver recipient, one cardiothoracic recipient) were reported to have an episode of rejection, neither underwent biopsy.

**Table 7:** Maternal outcomes in liver and cardiothoracic transplant recipients.

Maternal outcomes	Liver transplant cohort (n = 62)	Cardiothoracic cohort (n = 14)
**Maternal death**	0 (0)	1 (7)
**Critical care admission**	12 (19)	4 (29)
*Duration of stay:*		
1–2 days	8 (67)	3 (75)
More than 2 days	4 (33)	1 (25)
**Episode of rejection**	1 (2)	2 (14)
**Caesarean section**	27 (47)	8 (62)
*Grade of urgency* 1 *:*		
Grade 1–2	12 (46)	2 (25)
Grade 3–4	14 (54)	6 (75)
**Renal function during pregnancy**		
Highest serum creatinine >150 umol/l	5 (8)	4 (29)
Highest serum creatinine >125 umol/l	10 (16)	5 (36)
Highest serum creatinine >100 umol/l	20 (32)	11 (79)
More than 30% increase in serum creatinine	12 (19)	5 (36)
More than 20% increase in serum creatinine	21 (34)	9 (64)
**Blood pressure during pregnancy**		
Highest systolic blood pressure >160 mmHg	7 (11)	0 (0)
Highest diastolic blood pressure >100 mmHg	10 (16)	3 (21)
**Conditions during pregnancy**		
Pre-eclampsia	8 (13)	2 (14)
Gestational diabetes	4 (6)	2 (14)

Data are shown as *n* (%), with percentages referring to complete data only.

1 Grade 1 involves an immediate threat to the life of the woman or fetus; Grade 2 involves maternal or fetal compromise which is not immediately life-threatening; Grade 3 involves a need for early delivery but no maternal or fetal compromise; Grade 4 requires delivery at a time to suit the woman and maternity team [36].

Sixteen transplant recipients (21% of total; 12 liver recipients, 19%, four cardiothoracic recipients, 29%) were admitted to an intensive care (ITU) or high dependency unit (HDU), though this tended to be for a short duration (median 2 days, range 1–12 days).

Half of the cohort (n = 35) underwent caesarean section, with the majority classed as Grade 3–4 (59%, n = 20) urgency, where there was no immediate maternal or fetal compromise.

The most common indications for emergency caesarean delivery (urgency grade 1–2, 41%, n = 14) were fetal compromise (n = 9), including reduced fetal movements, cardiotocography abnormality and cord prolapse, and maternal compromise (n = 5), including pre-eclampsia and deteriorating graft function. Four women underwent non-emergency caesarean section solely due to their transplant or transplant surgery and two were at maternal request.

Ten women (13%) were diagnosed with pre-eclampsia during pregnancy and the percentage was similar between liver and cardiothoracic recipients. Six women were diagnosed with gestational diabetes, all of whom were on tacrolimus therapy throughout their pregnancy; four of the six women were also taking prednisolone.

Seventeen women (22%) were reported to have renal dysfunction during pregnancy with a 30% increase in serum creatinine and seven women (9%) had serum creatinine greater than 150 umol/l during the third trimester.

**Figure 2:**
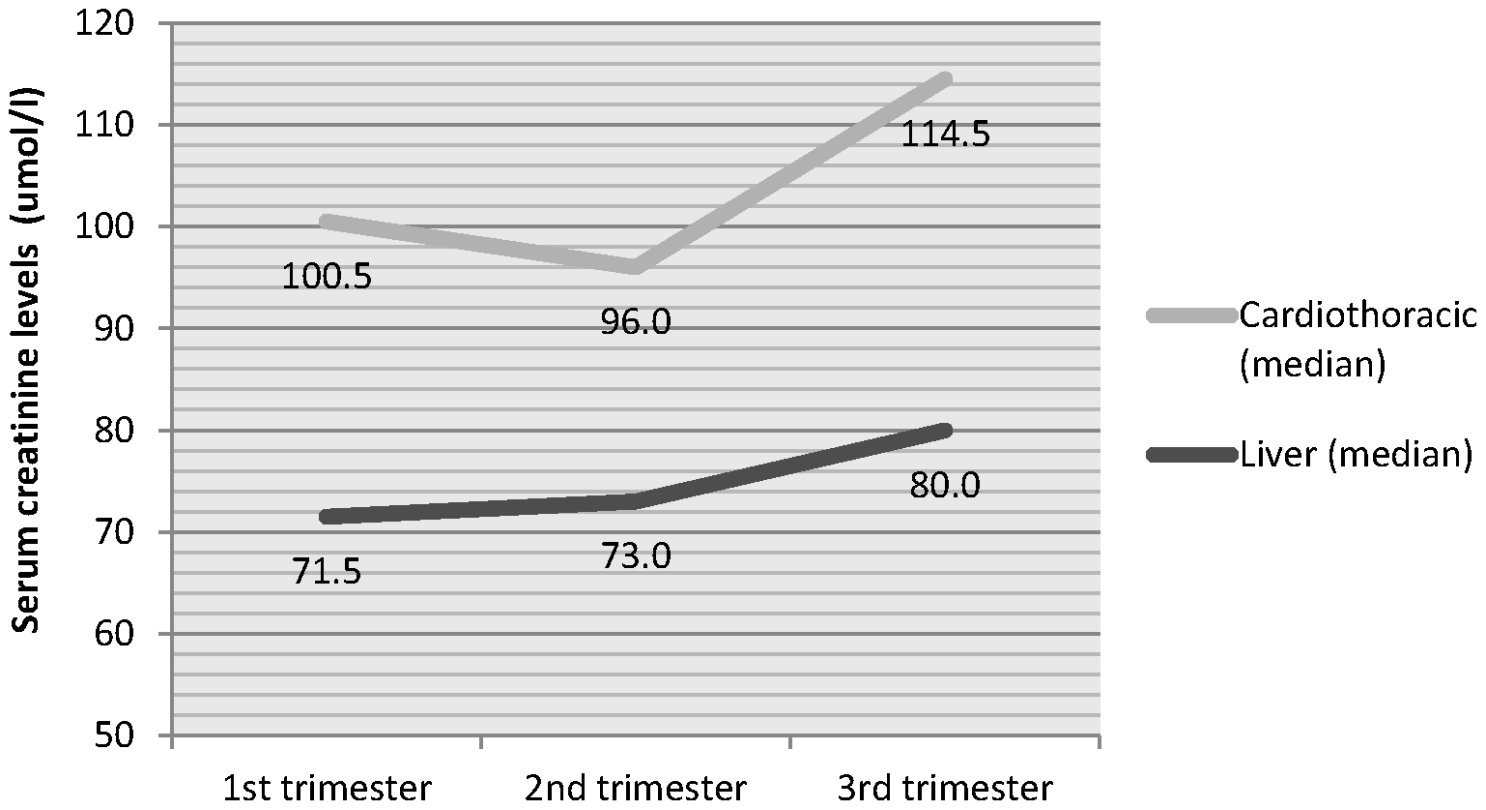
Highest serum creatinine level during each trimester of pregnancy, for liver and cardiothoracic transplant recipients.

Cardiothoracic transplant recipients had higher creatinine levels during pregnancy than liver transplant recipients, with mean serum creatinine of 104 during first trimester (vs. 77), and greater increases by the third trimester (see Figure 2). Creatinine did not decrease in the second trimester for liver transplant recipients.

Ten women (16%) in the liver transplant group had a diastolic blood pressure greater than 100 mmHg, whilst seven (11%) had a systolic blood pressure greater than 160 mmHg. Three women (21%) in the cardiothoracic group had a diastolic blood pressure of more than 100 mmHg, though none had a systolic blood pressure greater than 160 mmHg.

## Discussion

This study reports national, prospectively-collected pregnancy outcome data for UK liver and cardiothoracic transplant recipients, over a five year period. Similar to other studies [1], [10]–[12], we have found that the majority of pregnancies are successful in transplant recipients, but with a high rate of complications.

Liver transplant recipients, in comparison to cardiothoracic and renal transplant recipients from a separate UKOSS cohort [13], had similar livebirth rates (92% vs. 87% vs. 91%, respectively) but lower prematurity rates (42% vs. 54% vs. 52%), fewer low birthweight babies (37% vs. 54% vs. 48%), lower caesarean delivery rates (47% vs. 62% vs. 64%), similar maternal ICU admissions (19% vs. 29% vs. 21%) and fewer neonatal ICU admissions (25% vs. 54% vs. 38%). This same study found that a comparison cohort, of women from the general maternity population had higher livebirth rates (99%), lower prematurity rates (8%), fewer low birthweight babies (8%) and lower caesarean delivery rates (24%), further supporting the finding of a higher rate of complications in transplant recipients [13]. Thus complication rates in all transplant recipients are higher than in the general UK population, though liver recipients appear to have better rates than cardiothoracic and renal transplant recipients.

This is consistent with existing literature, in which more complications have been found in renal than liver transplant recipients, in both single-centre studies [14] and meta-analyses [10]. However, few studies have compared cardiothoracic transplant to other organ recipients [1], [3] and no meta-analyses exist.

While our study suggests poorer prognosis in the cardiothoracic group compared to our liver recipients, our cohort also had the lowest mean gestational age (35.5 weeks) and lowest mean birthweight (2441 g) when compared to the other cardiothoracic recipients in the literature (range 36.4–38.3 weeks, range 2600–2143 g) [6], [15]–[17]. As a national, prospective study we would expect our data to be less subject to selection bias and reporting bias inherent in single-centre studies and voluntary registries. Nonetheless, an important caveat to this finding is the comparatively small number of cases analysed, and no external source of case ascertainment was identified for our study period, as the UK Transplant Pregnancy Registry only covered 1994 to 2001.

In a national, retrospective study conducted in Sweden [18], which considered obstetric complications before and after organ transplantation, high rates were found in women who conceived in the years before transplant, particularly in renal compared to liver transplant recipients, suggesting the important role of pre-existing disease in affecting outcomes, particularly chronic kidney disease and hypertension [19]. These factors are likely to be applicable to our cardiothoracic cohort due to the high prevalence of moderately severe, pre-existing renal impairment, as evidenced by high creatinine levels (Figure 2), and congenital disease in this group of women (Table 2). Though our study found 21% (n = 3) of cardiothoracic recipients had diastolic blood pressure over 100 mmHg, other studies of heart transplant recipients and lung transplant recipients, specifically, have found rates of 39% and 52%, respectively [12], though it is not clear which thresholds for blood pressure or definition of “hypertension” they have used.

Another factor to consider is the generally poorer prognosis of cardiothoracic transplant recipients outside of pregnancy. National statistics have shown one-year survival in UK females of reproductive age (15–49 years), transplanted between 2005 and 2007 for kidney, heart, heart-lung, lung and liver was 98–100%, 85%, 71%, 79%, 93% respectively (unpublished data, NHS Blood and Transplant). Five-year survival in UK females of reproductive age, transplanted between 2005 and 2007 for kidney, heart, heart-lung, lung and liver was 92–98%, 80%, 57%, 53% and 80% respectively. Thus, one-year survival and five-year survival are generally lowest in cardiothoracic transplant recipients, and worse in liver than renal transplant recipients, which will be partly related to chronic rejection in the form of bronchiolitis obliterans syndrome [20] and cardiac allograft vasculopathy [21] limiting graft and patient survival after lung or cardiac transplantation, respectively, even in non-pregnant populations.

We cannot comment on whether the reasons women choose to become pregnant vary between regions within the UK or worldwide, and between transplant groups. It is possible that those women who became pregnant represent a healthier cohort than women who did not become pregnant, and this is a limitation that may distort results when making comparisons between groups.

### Allograft Function and Rejection in Pregnancy

Our study reports rejection rates of 2% in liver recipients (n = 1) and 14% in cardiothoracic recipients (n = 2). This was biopsy-proven in one of the cardiothoracic recipients, who died as a result of acute rejection. There were no other graft losses or biopsies undertaken. The UKOSS study of renal transplant recipients found 2% (n = 2) had rejection episodes [13].

Other studies have reported higher rates of rejection in liver recipients. For example, in a UK-based study, Christopher et al. [22] found 17% (n = 12) had rejection episodes during pregnancy, with an additional two cases (3%) occurring post-partum. Nagy et al. [23], in the USA, found 10.5% (n = 4) experienced rejection during pregnancy and a further two (5%) post-partum. In both studies, there were no graft losses or re-transplantations during pregnancy. Both studies were single-centre studies, conducted in transplant units. A survey-based study of female solid organ transplant recipients in British Columbia, Canada, found that 21% (n = 7) experienced a rejection episode [24].

The National Transplantation Pregnancy Registry (NTPR) found rejection rates of 16% in lung transplant recipients (n = 5), 0% in 5 heart-lung transplant recipients and 11% in heart transplant recipients (n = 11), with graft loss within 2 years of pregnancy of 3%, 20% and 14%, respectively [12]. A case series of cystic fibrosis lung transplant recipients found a particularly high rate of rejection (40%, n = 4), with progressive graft dysfunction resulting in death in all four women within 38 months of delivery [25].

Of note, two recent case reports [26], [27] document pregnancy-related sensitisation to HLA antigens, leading to rejection and graft failure in cardiac transplant recipients. One of the cases required re-transplantation (five months post-partum) [26], whilst the other died two years later [27]. These case reports highlight that although cardiothoracic recipients are at increased risk of graft loss and have lower survival rates, further research to explore the role of anti-HLA antibodies is needed [6].

### Medication at Conception and during Pregnancy

Evidence about the potential effects on pregnancy of the older immunosuppressive drugs is well established [28], however, there is less experience with some of the newer medications in pregnancy. Our study adds to the growing body of evidence that mycophenolate mofetil can lead to adverse fetal outcomes including congenital anomalies and a high probability of fetal loss [29].

Congenital anomalies most commonly associated with “mycophenolate embryopathy” include microtia and orofacial cleft defects [30], though there remain questions regarding the role of complex immunosuppressant regimens and interactions. None of these specific anomalies were reported in our cohort.

Of note, one of two patients to receive mycophenolate, with no adverse fetal outcome, was treated with anticoagulants and anti-platelet agents throughout pregnancy. The group receiving aspirin at conception had a statistically significant lower likelihood of adverse fetal outcomes, which is consistent with a recent meta-analysis considering perinatal death, growth restriction and preterm birth [31].

Only one patient in our study had exposure to sirolimus; she had a poor pregnancy outcome. Though there have been reports of successful pregnancies with sirolimus (16 of 23 pregnancies resulted in livebirths in one report [12]), uncertainty remains regarding potential teratogenic effects [32]. Interestingly, an earlier report from the NTPR found no livebirths in women who had continued on sirolimus throughout pregnancy, but successful fetal outcomes in those discontinuing during pregnancy [33]. It is important to note that most transplant recipients were receiving more than one medication and this may affect interpretation of the role of each medication in contributing to outcomes; a caveat in nearly all obvservational studies of pregnancy in women with complex diseases.

While the majority (60%) of our cohort breastfed their infants and current international consensus suggests it should not be viewed as absolutely contraindicated [5], the topic remains controversial and many centres advocate avoidance to their patients [15], [17]. Recent evidence suggests that tacrolimus therapy should not be a contraindication to breast feeding [34], [35]. The role of registries, such as the NTPR, will be integral in long-term follow-up of offspring for any adverse events.

## Conclusion

In common with most of the literature, our study found the majority of pregnancies in liver and cardiothoracic transplant recipients were successful, although there were high complication rates. Liver transplant recipients appear to have a better prognosis than both renal and cardiothoracic recipients, which may be related to them having a lower incidence of renal dysfunction, hypertension, congenital diseases and graft loss. This study confirmed the impact of renal dysfunction on pregnancy outcomes and the need for ongoing monitoring throughout pregnancy. We found an association between mycophenolate mofetil and poor fetal outcomes. Given the risks of graft rejection on maternal survival, this emphasises the role of pre-conception counselling in addressing these risks. Further research will be needed to investigate the long-term effects of pregnancy on maternal and graft survival rates, for which surveillance systems and national registries will prove invaluable.
